# Transcriptomic and proteomic analyses of SH-SY5Y neuroblastoma cells treated with amisulpride

**DOI:** 10.1017/neu.2025.10040

**Published:** 2025-09-16

**Authors:** Tsung-Ming Hu, Hsin-Yao Tsai, Shih-Hsin Hsu, Min-Chih Cheng

**Affiliations:** 1 Department of Psychiatry, Taipei Veterans General Hospital Yuli Branch, Hualien, Taiwan; 2 Department of Management, Fo Guang University, Jiaosi, Yilan, Taiwan

**Keywords:** Amisulpride, schizophrenia, SH-SY5Y, RNA sequencing, LC-MS/MS

## Abstract

**Objective:**

Amisulpride, a substituted benzamide derivative, has a unique pharmacological profile characterised by a high affinity for dopaminergic D_2_/D_3_ receptors, as well as an affinity for 5-HT_7_ receptors. Its effectiveness and safety surpass those of traditional antipsychotic drugs and multi-receptor antipsychotic medications in improving global symptoms, including both positive and negative symptoms. This makes it a compelling subject for study. However, the molecular mechanisms that contribute to its clinical efficacy in treating schizophrenia remain largely unexplored.

**Methods:**

We assessed cell viability following amisulpride treatment using the MTT and a real-time cell viability assay. Subsequently, we conducted RNA-seq and LC-MS/MS analyses to identify differentially expressed genes and proteins in SH-SY5Y neuroblastoma cells treated with amisulpride.

**Results:**

In the present study, we used RNA-seq analysis to identify downregulated expression of a transcriptional factor, *FOSB*, in amisulpride-treated SH-SY5Y neuroblastoma cells, while using LC-MS/MS analysis to identify multiple differentially expressed proteins in these cells. Among these differentially expressed proteins, we confirmed four proteins (ACTG1, ANP32E, CLTC, IPO8) that are differentially expressed under the administration of amisulpride.

**Conclusion:**

Our data reveal novel insights into the role of amisulpride in modulating the differential expression of genes and proteins. These findings, which involve genes/proteins related to AP-1 transcription factor family gene regulation, cytoskeleton, histone binding activity, the intracellular trafficking of receptors and endocytosis of a variety of macromolecules, and nuclear localisation signal, are particularly significant as they shed light on the molecular underpinnings of the clinical efficacy of amisulpride and the pathogenesis of schizophrenia.

## Significant outcomes

- Identifying differentially expressed genes and proteins enhances our understanding of the molecular mechanisms behind amisulpride’s clinical effects.
- It offers potentially new pathogenic mechanisms and treatment targets for schizophrenia.

## Limitations

- The sample size is small and was conducted using only the SH-SY5Y cell line.
- Other genes and proteins need to be verified to gain more insight into the molecular mechanism of amisulpride.

## Introduction

The effectiveness of traditional antipsychotic medications is linked to their ability to block dopamine D_2_ receptors (Willner, 1997). Haloperidol is a traditional antipsychotic drug that acts as a complete dopamine D_2_ receptor antagonist. It is effective in alleviating schizophrenia symptoms but may cause side effects, including extrapyramidal symptoms and elevated prolactin levels (Beasley *et al*., 1999). A broader spectrum of therapeutic efficacy of atypical antipsychotic drugs such as risperidone, olanzapine, and clozapine has been introduced to the market for treating schizophrenia (Newcomer, 2005). An atypical antipsychotic drug known as amisulpride is a substituted benzamide derivative that possesses a unique pharmacological profile, characterised by a high affinity for dopaminergic D_2_/D_3_ and an affinity for 5-HT_7_ receptors (Perrault *et al*., 1997; Moller, 2003). Amisulpride is more effective and safer than traditional and multi-receptor antipsychotics in improving global symptoms, including both positive and negative symptoms of schizophrenia (Komossa *et al*., 2010). Currently, there is still a limited understanding of the molecular mechanisms that contribute to the clinical effectiveness of amisulpride in treating schizophrenia.

Proteogenomics is a powerful strategy for gene and protein expression profiles in neuroscience and neurology (Nesvizhskii, 2014). For example, transcriptomic and proteomic analyses of antipsychotic drugs have identified genes associated with schizophrenia. (Bortolasci *et al*., 2020; Truong *et al*., 2022). A recent genome-wide mRNA expression study showed that eight antipsychotic drugs downregulate the expression of genes related to the focal adhesions pathway (Panizzutti *et al*., 2025). This suggests that adhesion pathways may play a role in the pathophysiology of bipolar disorder and schizophrenia.

However, many detailed molecular mechanisms of amisulpride’s action remain unknown. In this study, we performed RNA sequencing (RNA-seq) and LC-MS/MS analysis to identify the differentially expressed genes (DEGs) and proteins in SH-SY5Y neuroblastoma cells treated with amisulpride. A more precise understanding of the molecular effects of amisulpride on SH-SY5Y cells could offer insights into the molecular mechanisms underlying psychiatric disorders, potentially revealing novel treatment targets.

## Materials and methods

### Cell culture and amisulpride treatment

The human SH-SY5Y neuroblastoma cell line (Sigma catalogue no. 94030304) was cultured in Dulbecco’s Modified Eagle’s Medium (DMEM), supplemented with 10% fetal bovine serum (FBS), 100 units/ml penicillin, 100 µg/ml streptomycin, and 2 mM L-glutamine. The SH-SY5Y cells were cultured in a humidified environment with 5% CO_2_ at 37°C, and the medium was refreshed every two to three days. Amisulpride was sourced from Sigma–Aldrich (A2729). A stock solution was made in dimethyl sulfoxide (DMSO) and subsequently diluted in the medium to achieve the desired final concentration. The concentrations of amisulpride used in this study were selected based on previous research examining its effects on the SH-SY5Y cells (Park *et al*., 2011). We administered doses of 0.4, 4, 20, and 40 μg/ml, which are within a pharmacologically relevant range known to modulate the activity of D_2_/D_3_ and 5-HT_7_ receptors without causing cytotoxicity. A 24–hour treatment period was chosen to allow sufficient time for amisulpride-induced changes in the transcriptome and proteome while minimising downstream secondary stress responses or cell death. To ensure statistical robustness and reproducibility of the transcriptomic and proteomic data, we performed three independent biological replicates per condition, with each set derived from separate cell culture passages.

### MTT assay

The SH-SY5Y cells were cultured in 96-well plates at a density of 1.5 × 10^4^ cells per well and were incubated for 24 h. After this initial incubation, the cells were treated with amisulpride at various concentrations for 24 h in serum-free DMEM. Following treatment, the cells were washed twice with phosphate-buffered saline (PBS) and then cultured in DMEM supplemented with 10% FBS for two days. Subsequently, the cells were incubated with 0.5 mg/ml of the MTT (Sigma Chemical Co., St Louis, MO, USA) in DMEM for 4 h. Viable cells converted MTT to formazan, which appears blue-purple when dissolved in DMSO. The intensity of the colour, measured as absorbance, is directly proportional to the number of live cells. Absorbance at 545 nm was recorded using a microplate reader (Varioskan Flash, Thermo Fisher Scientific, Vantaa, Finland). The percentage of cell survival was calculated by dividing the absorbance of the amisulpride-treated samples by the absorbance of the corresponding DMSO-treated controls.

### Real-time cell viability assay

Real-time cell viability was conducted using the RealTime-Glo™ MT Cell Viability Assay (Cat.# G9711, Promega). The SH-SY5Y cells (1 × 10^4^ per well) were plated in a 96-well plate and cultured in a 5% CO_2_ atmosphere at 37°C overnight to ensure thorough incubation conditions. The medium was then replaced with a growth medium containing, various dosages of amisulpride, the MT Cell Viability Substrate (Promega) and NanoLuc Enzyme (Promega), and the cells were cultured for 72 h. Luciferase activity was measured using a microplate reader (VANTAstar^TM^, BMG LABTECH, Germany).

### Total RNA preparation, RNA-seq, and RT-qPCR

Total RNA preparation, RNA-seq, RT-qPCR, and DEG identification were performed following the methods described in a previous study (Wang *et al*., 2022). The target gene, *FOSB* (Hs00171851_m1, FAM™ dye-labelled TaqMan™ MGB probe), and two endogenous genes, *GAPDH* (Hs02786624_g1, VIC™ dye-labelled TaqMan™ MGB probe) and *18S* (Hs99999901_s1, VIC™ dye-labelled TaqMan™ MGB probe), were analysed using TaqMan™ gene expression assays according to the manufacturer’s protocol (ThermoFisher Scientific Inc.). The expression levels of *ACTG1*, *ANP32E*, *CLTC*, and *IPO8* were assayed using SYBR Green detection (ThermoFisher Scientific Inc.), and the primer sequences are listed in the Supplementary Table S1. All tests were performed in six replicates. Statistically significant differences between the treated and control groups were determined using a *p–*value threshold of <0.05.

### Protein sample preparation and LC-MS/MS analysis

To prepare protein samples, cells were washed twice with cold PBS and then resuspended in a lysis buffer that contain 20 mM HEPES (pH 7.6), 7.5-mM NaCl, 2.5 mM MgCl_2_, 0.1 mM EDTA, 0.1% TritonX-100, 50 mM NaF, 0.1 mM Na_3_VO_4_, and a protease inhibitor cocktail (one mini-tablet/10 ml; Roche Diagnostics GmbH). The homogenates were centrifuged at 13,000 r.p.m. for 30 minutes at 4°C, and the resulting supernatants were stored at -80°C until needed.

In the process of solution digestion, LC-MS/MS and protein identification were conducted by BIOTOOLS CO., LTD in Taiwan. The procedure began with diluting the protein solutions in 50 mM ammonium bicarbonate (ABC, Sigma). The samples were then reduced with 10 mM dithiothreitol (DTT, Merck) at 56°C for 45 minutes. Following this, cysteine residues were blocked with 50 mM iodoacetamide (IAM, Sigma) at 25°C for 30 minutes. Next, the samples were digested with sequencing-grade modified porcine trypsin (Promega) at 37°C for 16 h. After digestion, the peptides were desalted, dried by vacuum centrifugation, and stored at -80°C until further use.

In the meticulous LC-MS/MS analysis process, the digested peptides were diluted in HPLC buffer A (0.1% formic acid) and loaded onto a reverse-phase column (Zorbax 300 SB-C18, 0.3 × 5 mm; Agilent Technologies). The desalted peptides were separated using a column (Waters BEH 1.7 µm, 100 μm I.D. × 10 cm with a 15 μm tip) and a multi-step gradient of HPLC buffer B (99.9% acetonitrile/0.1% formic acid) over a period of 70 minutes, at a flow rate of 0.3 μl/min. The liquid chromatography apparatus was coupled with a 2D linear ion trap mass spectrometer (Orbitrap Elite ETD; Thermo Fisher), which was operated using Xcalibur 2.2 software (Thermo Fisher). The full-scan MS was performed in the Orbitrap over a range of 400 to 2,000 Da, with a a resolution of 120,000 at m/z 400. Internal calibration was achieved using the ion signal of protonated dodecamethylcyclohexasiloxane ion at m/z 536.165365 as the lock mass. The analysis included 20 data-dependent MS/MS scan events, each followed by one MS scan for the 20 most abundant precursor ions in the preview MS scan. The m/z values selected for MS/MS were dynamically excluded for 40 s with a relative mass window of 15 ppm. The electrospray voltage was set to 2.0 kV, and the capillary temperature was maintained at 200°C. Automatic gain control for MS and MS/MS was set to 1,000 ms (for full scan) and 200 ms (for MS/MS), or 3 × 10^6^ ions (full scan) and 3,000 ions (MS/MS) for maximum accumulated time or ions, respectively.

The protein identification was conducted using Proteome Discoverer software (version 2.3, Thermo Fisher Scientific). The MS/MS spectra were searched against the UniProt database utilising the Mascot search engine (Matrix Science, London, UK; version 2.5). For peptide identification, a mass tolerance of 10 ppm was allowed for intact peptide masses and 0.5 Da for CID fragment ions, with allowance for two missed cleavages resulting from the trypsin digestion. The variable modifications included oxidised methionine and acetylation at the protein N-terminal, while carbamidomethylation of cysteine was used as a static modification. Peptide-spectrum matches (PSM) were filtered based on high confidence, and the Mascot search engine ranked the top identification for each peptide, ensuring that the overall false discovery rate below 0.01. Proteins identified by only a single peptide hit were excluded from the final results.

### Bioinformatic analysis

For Gene Ontology (GO) enrichment and pathway analysis, differentially expressed proteins between amisulpride-treated and non-treated samples (*P* < 0.05) were subjected to the Database for Annotation, Visualisation, and Integrated Discovery (DAVID, https://davidbioinformatics.nih.gov/) using the official gene symbol method.

### Immunoblotting

Immunoblotting was conducted following standard protocols, utilising the primary antibodies listed below. Rabbit anti-ACTG1 (11227–1-AP; Proteintech, Rosemont, IL, USA), rabbit anti-ANP32E (A17220; ABclonal, Woburn, MA, USA), rabbit anti-CLTC (A12423; ABclonal), rabbit anti-CRMP1 (A2705; ABclonal), rabbit anti-HSD17B10 (A5448; ABclonal), rabbit anti-IPO8 (A14679; ABclonal), rabbit anti-NPEPPS (A08129–2; Boster Biological Technology, Pleasanton, CA, USA), rabbit anti-OTUB1 (A11656; ABclonal), rabbit anti-PRMT1 (A1055; ABclonal), rabbit anti-RAC1 (A7720; ABclonal), rabbit anti-SPTAN1 (A0160; ABclonal), rabbit anti-SEPTIN11 (A12189; ABclonal), rabbit anti-TIA1 (A12523; ABclonal), and mouse anti-GAPDH (G8795; Sigma–Aldrich, Saint Louis, MO, USA). Horseradish peroxidase-conjugated donkey anti-rabbit IgG (NA934V, GE Healthcare Life Sciences, UK) and human anti-mouse IgG (5220–0341; KPL) were used as secondary antibodies. Chemiluminescence was visualised using an enhanced chemiluminescence detection system (GTX400006; GeneTex).

## Results

### Effects of amisulpride on SH-SY5Y cell viability

**Figure 1.**
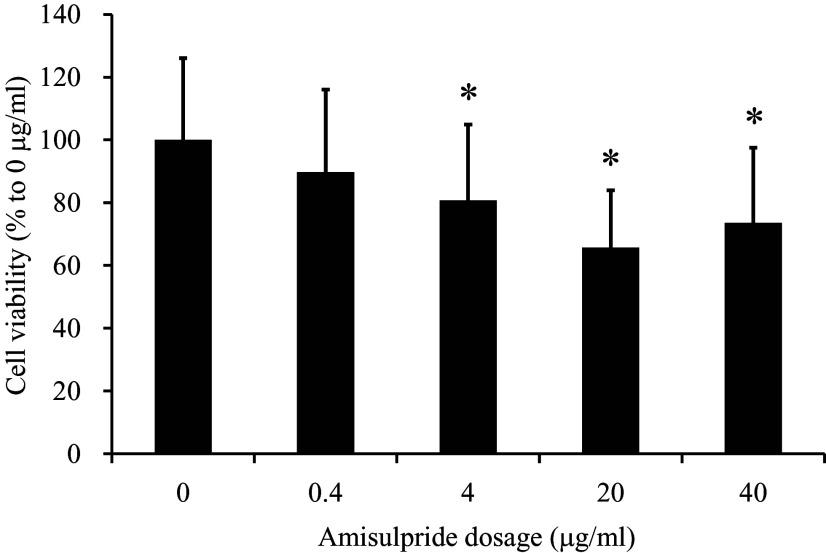
Amisulpride exhibits concentration-dependent effects on the viability of SH-SY5Y cells. The MTT assay was conducted to estimate cell numbers after a 24–hour treatment with varying concentrations of amisulpride. The results were calculated as the optical density at 545 nm (OD545) of amisulpride-treated cultures compared to non-treated control cultures and expressed as means ± standard deviation. A statistically significant difference between amisulpride-treated and non-treated cultures was identified using ANOVA, with post hoc tests performed using by LSD method (*p < 0.01, *n* = 80).

The effects of a 24–hour amisulpride treatment (0.4, 4, 20, and 40 μg/ml) on SH-SY5Y cell viability were examined using the MTT assay (Fig. 1). The survival rates compared to 0 μg/ml controls were 81% (*p* = 3E-06) after exposure to 4 μg/ml amisulpride, 66% (*p* = 1.4E-17) after exposure to 20 μg/ml, and 74% (*p* = 4.1E-09) after exposure to 40 μg/ml amisulpride.

**Figure 2.**
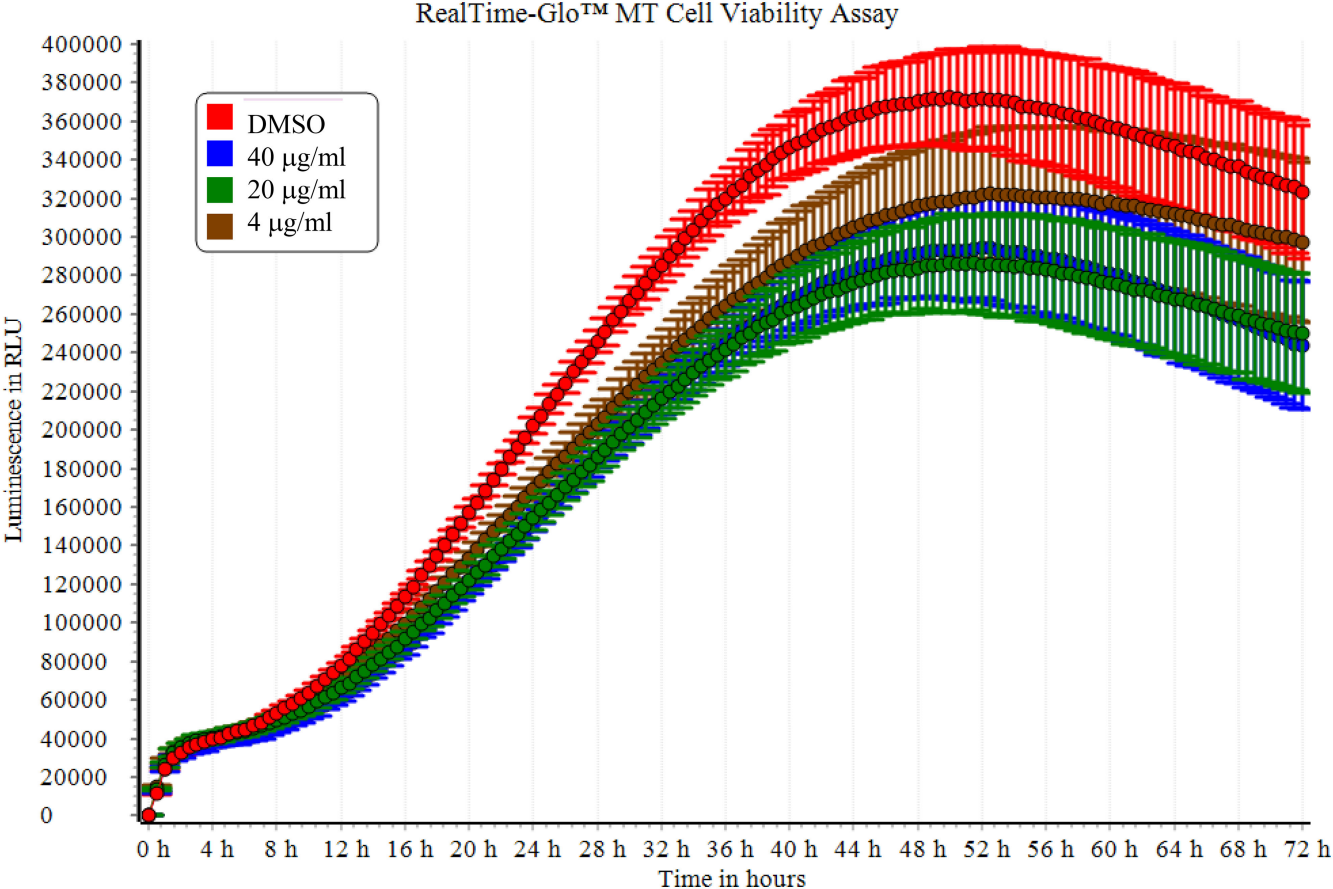
Analysis of amisulpride-treated SH-SY5Y cells with RealTime-Glo™ MT Cell Viability. Real-time cell viability (luminescent signals) was monitored every 30 minutes for 72 h on a VANTAstar^TM^ microplate reader with a gas control module (37°C and 5% CO_2_) (*n* = 6).

We measured cell viability over the 72 h of amisulpride treatment using the RealTime-Glo™ MT Cell Viability Assay. Our results confirmed that amisulpride at concentrations of 4, 20, and 40 μg/ml) inhibited cell survival compared to the 0 μg/ml control group (Fig. 2).

### Identification of differentially expressed genes by transcriptome sequencing

**Figure 3.**
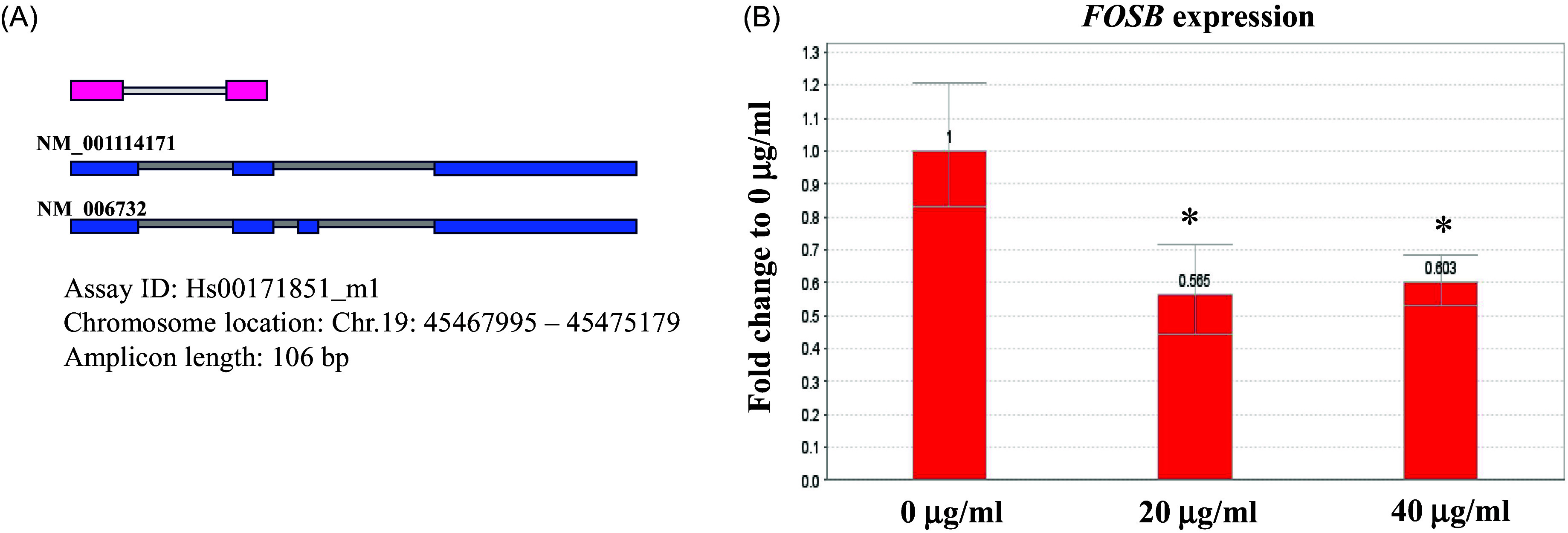
The mRNA expression level of *FOSB* in amisulpride-treated SH-SY5Y cells. (A) a schematic representation of the *FOSB* gene genomic map, including primers and probes (pink rectangle) used for the TaqMan assay (Hs00171851_m1 from Thermo Fisher Scientific). (B) RT-qPCR assay results showing the expression of *FOSB* in SH-SY5Y cells treated with amisulpride. GAPD*H* was used as an endogenous gene for normalisation. Data are expressed as fold changes relative to 0 μg/ml of amisulpride ± standard deviation (* *p* < 0.05; *n* = 6).

Nine samples from three biological replicates (with doses of 0, 20, and 40 ug/ml of amisulpride) were used for transcriptome sequencing. The number of reads per sample ranged from 38,707,688 to 51,321,892 across the nine sequenced RNA samples (Supplementary Table S2). Differential gene expression analysis revealed that the *FOSB* gene was significantly downregulated in the group treated with 20 μg/ml amisulpride group, with a fold change greater than 2 and an adjusted *p*-value of 7.46E-11 (Supplementary Table S3 and S4). The mRNA expression level of *FOSB* was confirmed in biologically replicated SH-SY5Y cells using a real-time quantitative PCR (RT-qPCR) assay (Fig. 3).

### Protein ID, bioinformatic analysis, immunoblot verification, and RT-qPCR analysis

We utilised a label-free LC-MS/MS shotgun proteomics strategy to identify proteins that were differentially expressed in the SH-SY5Y cells treated with amisulpride. We analysed nine samples from three biological replicates at three different doses of amisulpride: 0, 20, and 40 μg/ml. Normalised peptide-spectrum matches (PSMs) were calculated using the following formula: (PSM in sample A/total PSM in sample A) × average PSM across all nine samples. Supplementary Table S5 lists the protein accession numbers, descriptions, and normalised PSMs of the differentially expressed proteins between amisulpride-treated and non-treated samples. To evaluate the differences in normalised PSMs between the two groups, we conducted Student’s t-tests with a significance threshold of *P* < 0.05.

**Figure 4.**
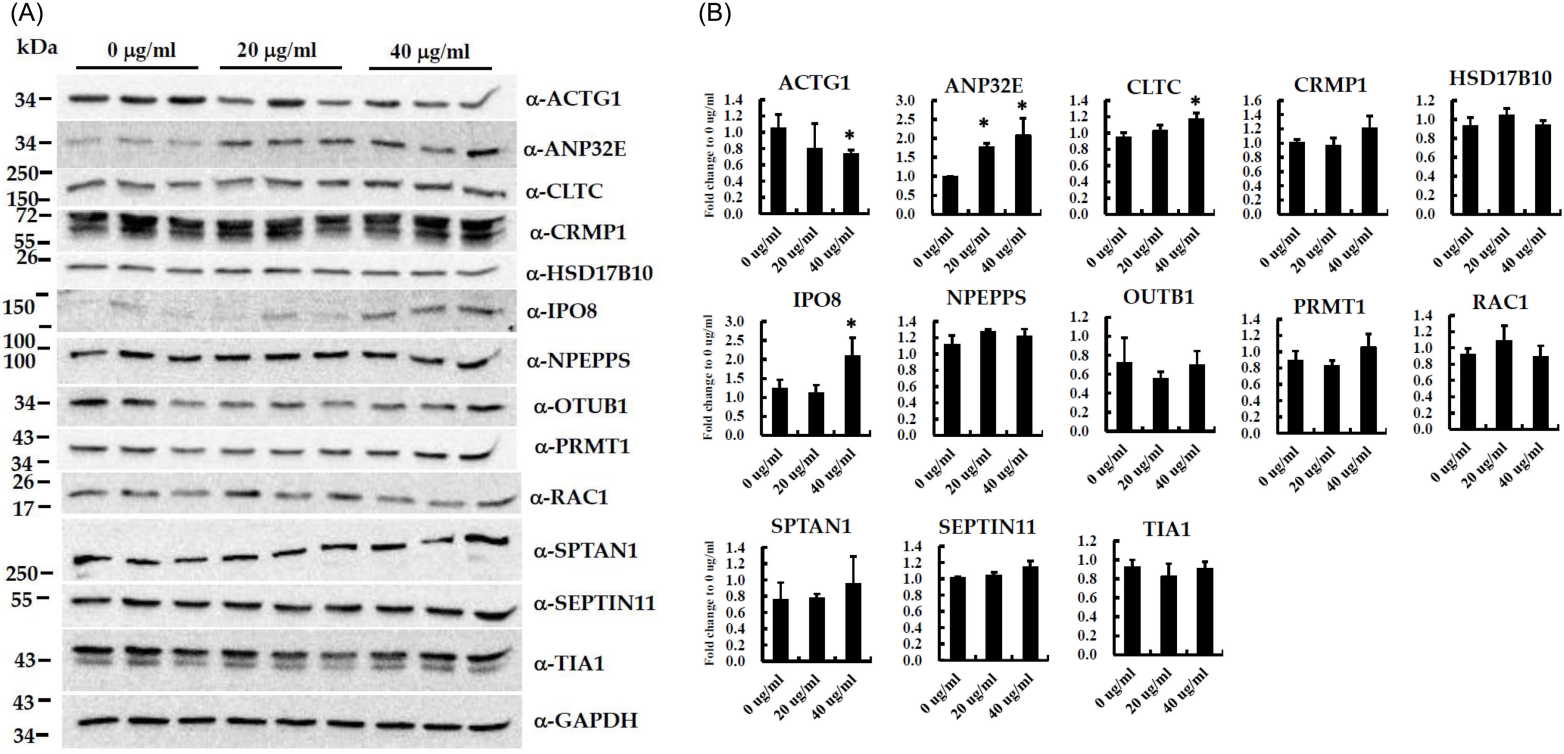
Immunoblotting analysis to validate the differential expression for 13 proteins in amisulpride-treated biological replicated SH-SY5Y cells. (A) immunoblotting and (B) quantification of protein expression showing the fold differences of four proteins (ACTG1, ANP32E, CLTC, IPO8) between amisulpride-treated groups and control. GAPDH was a loading control. Data are expressed as fold change to 0 ug/ml of amisulpride ± standard deviation (*p < 0.01, *n* = 3).

GO enrichment and pathway analysis revealed that the differentially expressed proteins between amisulpride-treated and non-treated samples were significantly associated with several GO terms and three pathways after applying the Bonferroni adjustment (Table 1, P<0.05). Thirteen proteins that exhibited differential expression between the groups were selected for further verification using immunoblot analysis on independent biological replicates of the SH-SY5Y cells (Fig. 4A). We determined the fold differences in the expression of the selected proteins (Fig. 4B), and confirmed the differential expression of four proteins: ACTG1, ANP32E, CLTC, and IPO8. We compared the mRNA expression levels of four genes (*ACTG1*, *ANP32E*, *CLTC*, and *IPO8*) in biologically replicated SH-SY5Y cells treated with amisulpride (0, 20, and 40 μg/ml) using RT-qPCR assay. We did not find significant differences in expression among these four genes across the three groups (Supplementary Figure S1).

**Table 1.** Summary of GO enrichment and pathway analyses in differentially expressed proteins between amisulpride-treated and non-treated samples

*Category*	*Term*	*Protein count*	*Protein IDs*	*Bonferroni P-value*
BP	GO:0002181∼cytoplasmic translation	7	*RPS14, RPL5, RPL32, RPL35A, RPL35, RPS10, RPL6*	3.22E−04
BP	GO:0006412∼translation	8	*RPS14, RPL5, RPL32, NARS1, RPL35A, RPL35, RPS10, RPL6*	0.0054
BP	GO:0006418∼tRNA aminoacylation for protein translation	4	*GARS1, NARS1, LARS1, IARS1*	0.0348
CC	GO:0005737∼cytoplasm	50	*RPL5, RPL32, GDI2, HMGB2, NUCKS1, YBX1, ACTB, RPL6, ACTG1, EEF1B2, NPEPPS, RPS14, NARS1, SCP2, RPL35, RAC1, SPTAN1, RPS10, ANXA2, PRMT1, ACTN1, CTPS1, ACTN4, TUBA4B, DNM2, ACTA1, LARS1, PSME3, ARL3, PHYHIPL, CRMP1, HSD17B10, MYG1, HNRNPDL, PRKAR2B, CBS, DDT, IARS1, TIA1, ALYREF, HUWE1, RPL35A, NUDT21, GARS1, GLUD1, EIF5, COPS2, HNRNPA2B1, ANP32E, ADSS2*	6.83E-12
CC	GO:0070062∼extracellular exosome	32	*RPL5, ARL3, GDI2, CLTC, YBX1, ACTB, ACTG1, RPS14, NPEPPS, NARS1, PRKAR2B, DDT, PSMD2, IARS1, RAC1, ATP6V1E1, SPTAN1, TXNDC5, ANXA2, ALYREF, ACTN1, HUWE1, RPL35A, ACTN4, CYRIB, TUFM, DNM2, GARS1, ACTA1, HNRNPA2B1, ADSS2, OTUB1*	2.83E-11
CC	GO:0005829∼cytosol	46	*RPL5, RPL32, USO1, GDI2, CLTC, CRMP1, YBX1, RPL6, ACTB, ACTG1, EEF1B2, RPS14, NPEPPS, IPO8, HNRNPDL, NARS1, SCP2, RBBP4, PRKAR2B, CBS, PSMD2, RPL35, IARS1, RAC1, ATP6V1E1, ENOPH1, SPTAN1, RPS10, TIA1, USP5, ANXA2, PRMT1, ALYREF, ACTN1, HUWE1, RPL35A, CTPS1, DNM2, GARS1, ACTA1, EIF5, LARS1, PSME3, COPS2, ADSS2, OTUB1*	1.52E-09
CC	GO:1990904∼ribonucleoprotein complex	12	*RPS14, RPL5, TIA1, RPL32, HNRNPDL, HNRNPA2B1, RPL35A, ACTN4, YBX1, RPS10, RPL6, ACTB*	4.36E-07
CC	GO:0005925∼focal adhesion	12	*RPS14, RPL5, ACTN1, GDI2, CLTC, ACTN4, RAC1, RPS10, RPL6, ACTB, ACTG1, DNM2*	2.50E-05
CC	GO:0022626∼cytosolic ribosome	7	*RPS14, RPL5, RPL32, RPL35A, RPL35, RPS10, RPL6*	1.48E-04
CC	GO:0022625∼cytosolic large ribosomal subunit	5	*RPL5, RPL32, RPL35A, RPL35, RPL6*	0.0074
CC	GO:0045202∼synapse	10	*EIF5, RPL32, GDI2, RPL35A, RAC1, YBX1, ACTB, TUFM, ACTG1, DNM2*	0.0098
MF	GO:0003723∼RNA binding	26	*RPL5, RPL32, USO1, GDI2, CLTC, HMGB2, NUCKS1, YBX1, HSD17B10, RPL6, RPS14, HNRNPDL, RPL35, EIF3CL, RPS10, TIA1, ANXA2, PRMT1, ALYREF, HUWE1, RPL35A, ACTN4, TUFM, NUDT21, EIF5, HNRNPA2B1*	1.56E-09
MF	GO:0005515∼protein binding	62	*RPL5, HIBADH, CLTC, GDI2, HMGB2, NUCKS1, YBX1, ACTB, SEPTIN11, RPL6, ACTG1, EEF1B2, RPS14, MYDGF, IPO8, NARS1, SCP2, PSMD2, EIF3CL, RAC1, ATP6V1E1, SPTAN1, RPS10, ENOPH1, USP5, ANXA2, PRMT1, ACTN1, CTPS1, ACTN4, TUFM, DNM2, ACTA1, LARS1, PSME3, HAT1, OTUB1, ARL3, USO1, PHYHIPL, CRMP1, HSD17B10, MYG1, HNRNPDL, RBBP4, PRKAR2B, CBS, IARS1, TXNDC5, TIA1, ALYREF, HUWE1, RPL35A, CYRIB, NUDT21, GARS1, GLUD1, EIF5, COPS2, HNRNPA2B1, ANP32E, ADSS2*	0.0053
MF	GO:0003735∼structural constituent of ribosome	7	*RPS14, RPL5, RPL32, RPL35A, RPL35, RPS10, RPL6*	0.0120
MF	GO:0005525∼GTP binding	9	*GLUD1, EIF5, ARL3, RAC1, ADSS2, TUBA4B, SEPTIN11, TUFM, DNM2*	0.0188
KEGG	hsa05100: Bacterial invasion of epithelial cells	6	*CLTC, RAC1, SEPTIN11, ACTB, ACTG1, DNM2*	0.0051
KEGG	hsa03010: Ribosome	7	*RPS14, RPL5, RPL32, RPL35A, RPL35, RPS10, RPL6*	0.0249
BIOCARTA	h_uCalpainPathway: uCalpain and friends in Cell spread	4	*ACTA1, ACTN1, RAC1, SPTAN1*	0.0270

BP: biological process; CC: cellular component; MF: molecular function; KEGG: Kyoto Encyclopaedia of Genes and Genomes (https://www.genome.jp/kegg/).

## Discussion

Antipsychotic medications are the preferred treatment of choice for patients experiencing psychotic symptoms, including delusions, hallucinations, bizarre behaviour, agitation, and aggression. Research indicates that changes in neural plasticity, driven by differential gene expression in the brain, may support the clinical effectiveness of antipsychotic medications (Hyman & Nestler, 1996). In this study, we conducted a transcriptomic analysis to discover a downregulated expression of a transcriptional factor, the *FOSB* gene, in SH-SY5Y neuroblastoma cells treated with amisulpride. We also performed proteomic analysis to identify several proteins differentially expressed in the amisulpride-treated cells, which involved pathways related to synapse, extracellular exosome, structural constituent of ribosome, RNA and protein binding, and bacterial invasion of epithelial cells. We confirmed four specific proteins (ACTG1, ANP32E, CLTC, and IPO8) among the differentially expressed proteins, which showed significant changes in expression due to amisulpride administration, using an immunoblotting assay.

The activator protein 1 (AP-1) transcription factor family is a crucial class of transcriptional regulators that control various aspects of cell physiology in response to environmental changes, including stress, cytokines, infections, oncogenic stimuli, and growth factors (Durchdewald *et al*., 2009). Upon regulation, the AP-1 controls the expression of target genes associated with cell proliferation, differentiation, transformation, apoptotic cell death, and migration (Hisanaga *et al*., 1990; Durchdewald *et al*., 2009). The AP-1 family includes essential region leucine zipper (bZIP) domain proteins such as JUN, FOS, and FOSB. These proteins must dimerise to form the transcription factor complex AP-1 before binding to their DNA target sites (Wagner, 2001). Research suggests that the transcriptional activation of the AP-1 complex plays a vital role in the central nervous system, brain development, and psychiatry disorders (Pennypacker, 1995). For example, multiple studies have reported changes in fos and jun family genes as markers of transcriptional activity alterations caused by antipsychotic drug treatment (Kontkanen *et al*., 2002; Kiss & Osacka, 2020). A study found that *FosB* mutant mice lost the induction of Fos-related proteins by chronic cocaine exposures and exhibited abnormal locomotor and conditioned place preference responses (Hiroi *et al*., 1997). In this study, we observed a reduction in the gene expression of the *FOSB* gene in the SH-SY5Y cells treated with amisulpride. This suggests that the *FOSB* gene may play a role in the molecular mechanism of antipsychotic drugs and the pathophysiology of psychiatric disorders. Notably, two studies have reported an association between the *FOSB* gene and the pathogenesis of schizophrenia, although their findings are inconsistent. A genome-wide gene expression study has found that the *FOSB* gene was upregulated in fibroblast samples from individuals with schizophrenia (Huang *et al*., 2019). In contrast, a transcriptomic study revealed that the *FOSB* gene is downregulated in the brain tissue of patients with schizophrenia (Chen *et al*., 2023). The discrepancy might be attributed to differences in antipsychotic treatment and the types of tissue collected. Taken together, we propose that amisulpride’s effects on the regulation of genes within the AP-1 transcription factor family are linked to mental illnesses that feature psychotic symptoms. Moreover, the *FOSB* gene was acceptable as a candidate gene for schizophrenia and may play a role in its pathogenesis.

Evidence indicates that abnormal synaptogenesis and synaptic dysfunction are crucial to the pathophysiology of schizophrenia, highlighting potential therapeutic targets for synaptic circuit modulation in psychiatric disorders (Howes & Onwordi, 2023; Wolf & Abi-Dargham, 2023). In this study, we found amisulpride treatment can increase the protein level of a synaptic vesicle-related protein, clathrin heavy chain (CLTC), in the SH-SY5Y cells. CLTC encodes clathrin heavy chain, a crucial component of clathrin-coated vesicles that mediate intracellular trafficking, especially endocytosis and membrane recycling (Narayana *et al*., 2019; Itagaki & Kamei, 2025). Recent studies have shown that CLTC is also involved in synaptic transmission and the cycling of synaptic vesicles (Pannone *et al*., 2023; Yang *et al*., 2025a). Furthermore, several studies indicate that *CLTC* mutations are associated with childhood-onset schizophrenia, neurodevelopmental disorders, epileptic encephalopathy, and Parkinsonism (DeMari *et al*., 2016; Manti *et al*., 2019, Nabais Sá et al., 2020, Usnich *et al*., 2024). Overall, dysfunction of CLTC may contribute to a wide range of neuropsychiatric risks. Therefore, CLTC could be a potential therapeutic target for treatment of psychiatric disorders.

Mounting evidence underscores the pivotal role of actin remodelling in synaptogenesis, synaptic plasticity, and the intricate development of neurites in burgeoning neurons (Matus, 2000; Hotulainen & Hoogenraad, 2010). For instance, the dynamic nature of actin filaments is instrumental in forming dendritic spines during development, and they contribute significantly to the structural plasticity of mature synapses (Matus, 2000). A growing body of research has illuminated the regulatory mechanisms that finely tune actin dynamics within dendritic spines (Mattila & Lappalainen, 2008; Hotulainen & Hoogenraad, 2010). Notably, Kimoto and colleagues discovered that levels of transcripts associated with actin and mitochondrial oxidative phosphorylation are profoundly altered in individuals with schizophrenia (Kimoto *et al*., 2022). Furthermore, a study reports alterations in the dendritic spine across multiple brain regions in schizophrenia (Glausier & Lewis, 2013). The compelling evidence suggests that the abnormal morphology of dendritic spines observed in schizophrenia may indeed be linked to disruptions in the delicate regulation of actin cytoskeletal dynamics. In this study, we found that amisulpride treatment can decrease the protein level of ACTG1 in the SH-SY5Y cells. The *ACTG1* gene encodes cytoplasmic gamma actin, which is likely to play a vital role in cell morphology, motility, and other actin-related functions (Rivière *et al*., 2012). In neurons, ACTG1 is involved in shaping dendritic spines and synaptic structures (Schreiber *et al*., 2015), indicating its contribution to learning, memory, and neuroplasticity by facilitating synaptic remodelling. Variants in the *ACTG1* gene were observed in patients with Baraitser-Winter syndrome and agenesis of the corpus callosum and neuronal heterotopia (Vontell *et al*., 2019). Besides, several reports show variants in the *ACTG1* gene are associated with obsessive–compulsive disorder (Göbel *et al*., 2022) and autism spectrum disorder (Tuncay *et al*., 2022). Therefore, the processes associated with *ACTG1* in actin cytoskeletal dynamics may represent novel therapeutic targets for schizophrenia. However, the relationship between the *ACTG1* gene and the pathogenesis of schizophrenia requires further detailed elucidation.

Research indicates that antipsychotic medications can alter epigenomic patterns, including DNA methylation and histone modifications, affecting gene expression in the brain (Marques *et al*., 2025). Understanding the biological functions of epigenetics in the field of psychiatry would facilitate the development of new therapies for psychiatric disorders. In this study, we found amisulpride treatment can increase the protein level of ANP32E in the SH-SY5Y cells. The *ANP32E* gene encodes acidic nuclear phosphoprotein 32 family member E, which enables histone binding, acts as a histone chaperone, and assists in protein folding (Obri *et al*., 2014). It plays important roles in regulating chromatin, controlling gene expression, and helping the cell response to DNA damage (Obri *et al*., 2014). Gilda Stefanelli discovered that ANP32E plays a crucial role in regulating memory formation, transcription, and dendritic morphology by controlling steady-state H2A.Z binding in neurons (Stefanelli *et al*., 2021). Schizophrenia is increasingly recognised as a neurodevelopmental disorder that involves epigenetic factors (Yang *et al*., 2025b). We hypothesise that ANP32E impacts which neuronal genes are active or silenced during brain development by regulating chromatin structure through the eviction of H2A.Z. Taken together, we propose that amisulpride’s action on ANP32E may be linked to the transcriptional regulation of target genes associated with histone chaperones, which provides potentially novel pathogenic mechanisms and treatment targets for psychiatric disorders.

The *IPO8* gene encodes importin-8, a nuclear transport belonging to the importin b family, which mediates the import of proteins into the nucleus (Görlich *et al*., 1997; Miyamoto *et al*., 2016). It plays crucial roles in nucleocytoplasmic trafficking, gene regulation, and signal transduction (Liang *et al*., 2013; Wei *et al*., 2014). A genetic study identified a single-nucleotide polymorphism in the *IPO8* gene that is associated with the brain systems responsible for eye movement, which are known to be impaired in psychotic disorders (Lencer *et al*., 2017). Notably, Nganou et al., demonstrated that *IPO8* knockdown was associated with defects in neuronal migration (Nganou *et al*., 2018). Numerous studies show that IPO8 is expressed in various tissues, including the adult brain, which is known to transport several proteins essential for brain development (Yao *et al*., 2008; Weinmann *et al*., 2009; Volpon *et al*., 2016). Based on the above evidence, we hypothesise that dysfunction of IPO8 could disrupt nucleocytoplasmic trafficking, resulting in abnormal transcriptional regulation of neuronal genes associated with schizophrenia. The up-regulation of IPO8 induced by amisulpride, as demonstrated in this study, may be relevant to the clinical efficacy of antipsychotic medications.

To summarise, our data suggest that amisulpride may influence the differential expression of genes and proteins related to the AP-1 transcription factor family, cytoskeleton, histone binding activity, intracellular trafficking of receptors, endocytosis of various macromolecules, and nuclear localisation signals. This understanding of the molecular mechanisms underlying the clinical effectiveness of amisulpride and the pathogenesis of schizophrenia opens up exciting possibilities for further research. The detailed transcriptomic and proteomic analysis of amisulpride treatment provides a comprehensive understanding of cellular response at the molecular level, sparking curiosity and the need for more in-depth studies in the field.

This study has a major limitation. SH-SY5Y cells may not be the ideal standalone model for schizophrenia research. Although SH-SY5Y cells are a human-derived neuroblastoma cell line widely used in neuroscience due to their catecholaminergic properties and their ability to differentiate into neuron-like cells, researchers often prefer to use primary neuronal cells, co-culture systems, and rodent models (Park *et al*., 2011; Shipley *et al*., 2016). These alternatives are more effective for investigating synaptic function, morphology, neurotoxicity, neurotransmitter release, and disease modelling. Consequently, the findings of this study should be interpreted with caution.

## Supporting information

Hu et al. supplementary material 1 Hu et al. supplementary material

Hu et al. supplementary material 2 Hu et al. supplementary material

Hu et al. supplementary material 3 Hu et al. supplementary material

Hu et al. supplementary material 4 Hu et al. supplementary material

Hu et al. supplementary material 5 Hu et al. supplementary material

Hu et al. supplementary material 6 Hu et al. supplementary material

## Supplementary material

The supplementary material for this article can be found at https://doi.org/10.1017/neu.2025.10040.

## References

[ref1] Beasley CM , Dellva MA , Tamura RN , Morgenstern H , Glazer WM , Ferguson K and Tollefson GD (1999) Randomised double-blind comparison of the incidence of tardive dyskinesia in patients with schizophrenia during long-term treatment with olanzapine or haloperidol. British Journal of Psychiatry 174, 23–30. 10.1192/bjp.174.1.23 10211147

[ref2] Bortolasci CC , Spolding B , Kidnapillai S , Connor T , Truong TTT , Liu ZSJ , Panizzutti B , Richardson MF , Gray L , Berk M , Dean OM and Walder K (2020) Transcriptional effects of psychoactive drugs on genes involved in neurogenesis. International Journal of Molecular Sciences 21, 8333. 33172123 10.3390/ijms21218333 PMC7672551

[ref3] Chen Y , Dai J , Tang L , Mikhailova T , Liang Q , Li M , Zhou J , Kopp RF , Weickert C , Chen C and Liu C (2023) Neuroimmune transcriptome changes in patient brains of psychiatric and neurological disorders. Mol Psychiatry 28, 710–721. 36424395 10.1038/s41380-022-01854-7 PMC9911365

[ref4] Demari J , Mroske C , Tang S , Nimeh J , Miller R and Lebel RR (2016) CLTC as a clinically novel gene associated with multiple malformations and developmental delay. American Journal of Medical Genetics Part A 170, 958–966. 10.1002/ajmg.a.37506 26822784

[ref5] Durchdewald M , Angel P and Hess J (2009) The transcription factor Fos: a Janus-type regulator in health and disease. Histology and Histopathology 24, 1451–1461. 19760594 10.14670/HH-24.1451

[ref6] Glausier JR and Lewis DA (2013) Dendritic spine pathology in schizophrenia. Neuroscience 251, 90–107. 22546337 10.1016/j.neuroscience.2012.04.044 PMC3413758

[ref7] Göbel T , Berninger L , Schlump A , Feige B , Runge K , Nickel K , Schiele MA , Van Elst LT , Hotz A , Alter S , Domschke K , Tzschach A and Endres D (2022) Obsessive–compulsive symptoms in ACTG1-associated Baraitser-Winter cerebrofrontofacial syndrome. Journal of Neural Transmission 129, 1387–1391. 36205783 10.1007/s00702-022-02544-y PMC9550762

[ref8] Görlich D , Dabrowski M , Bischoff FR , Kutay U , Bork P , Hartmann E , Prehn S and Izaurralde E (1997) A novel class of RanGTP binding proteins. Journal of Cell Biology 138, 65–80. 9214382 10.1083/jcb.138.1.65 PMC2139951

[ref9] Hiroi N , Brown JR , Haile CN , Ye H , Greenberg ME and Nestler EJ (1997) FosB mutant mice: loss of chronic cocaine induction of fos-related proteins and heightened sensitivity to cocaines psychomotor and rewarding effects. Proceedings of the National Academy of Sciences USA 94, 10397–10402. 10.1073/pnas.94.19.10397 PMC23374 9294222

[ref10] Hisanaga K , Sagar SM , Hicks KJ , Swanson RA and Sharp FR (1990) c-fos proto-oncogene expression in astrocytes associated with differentiation or proliferation but not depolarization. Molecular Brain Research 8, 69–75. 2166202 10.1016/0169-328x(90)90011-2

[ref11] Hotulainen P and Hoogenraad CC (2010) Actin in dendritic spines: connecting dynamics to function. Journal of Cell Biology 189, 619–629. 20457765 10.1083/jcb.201003008 PMC2872912

[ref12] Howes OD and Onwordi EC (2023) The synaptic hypothesis of schizophrenia version III: a master mechanism. Molecular Psychiatry 28, 1843–1856. 37041418 10.1038/s41380-023-02043-w PMC10575788

[ref13] Huang J , Liu F , Wang B , Tang H , Teng Z , Li L , Qiu Y , Wu H and Chen J (2019) Central and peripheral changes in FOS expression in schizophrenia based on genome-wide gene expression. Frontiers in Genetics 10, 232. 30967896 10.3389/fgene.2019.00232 PMC6439315

[ref14] Hyman SE and Nestler EJ (1996) Initiation and adaptation: a paradigm for understanding psychotropic drug action. American Journal of Psychiatry 153, 151–162. 8561194 10.1176/ajp.153.2.151

[ref15] Itagaki M and Kamei N (2025) Clathrin functions in intracellular sorting during receptor-mediated endocytosis. FEBS Letters 599, 1509–1517. 39973445 10.1002/1873-3468.70016

[ref16] Kimoto S , Hashimoto T , Berry KJ , Tsubomoto M , Yamaguchi Y , Enwright JF , Chen K , Kawabata R , Kikuchi M , Kishimoto T and Lewis DA (2022) Expression of actin- and oxidative phosphorylation-related transcripts across the cortical visuospatial working memory network in unaffected comparison and schizophrenia subjects. Neuropsychopharmacology 47, 2061–2070. 35034100 10.1038/s41386-022-01274-9 PMC9556568

[ref17] Kiss A and Osacka J (2020) c-Fos and FosB/ΔFosB colocalizations in selected forebrain structures after olanzapine, amisulpride, aripiprazole, and quetiapine single administration in rats preconditioned by two different mild stressors sequences. Endocrine Regulations 54, 43–52. 32597143 10.2478/enr-2020-0006

[ref18] Komossa K , Rummel-Kluge C , Hunger H , Schmid F , Schwarz S , Silveira Da Mota Neto JI , Kissling W and Leucht S (2010) Amisulpride versus other atypical antipsychotics for schizophrenia, Cd006624. *Cochrane Database System Review* 2010, Cd006624. 10.1002/14651858.CD006624.pub2 PMC4164462 20091599

[ref19] Kontkanen O , Lakso M , Wong G and Castren E (2002) Chronic antipsychotic drug treatment induces long-lasting expression of fos and jun family genes and activator protein 1 complex in the rat prefrontal cortex. Neuropsychopharmacology 27, 152–162. 12093589 10.1016/S0893-133X(02)00289-0

[ref20] Lencer R , Mills LJ , Alliey-Rodriguez N , Shafee R , Lee AM , Reilly JL , Sprenger A , Mcdowell JE , Mccarroll SA , Keshavan MS , Pearlson GD , Tamminga CA , Clementz BA , Gershon ES , Sweeney JA and Bishop JR (2017) Genome-wide association studies of smooth pursuit and antisaccade eye movements in psychotic disorders: findings from the B-SNIP study. Translational Psychiatry 7, e1249. 29064472 10.1038/tp.2017.210 PMC5682604

[ref21] Liang P , Zhang H , Wang G , Li S , Cong S , Luo Y and Zhang B (2013) KPNB1, XPO7 and IPO8 mediate the translocation ofNF-κB/p65 into the nucleus. Traffic 14, 1132–1143. 23906023 10.1111/tra.12097

[ref22] Manti F , Nardecchia F , Barresi S , Venditti M , Pizzi S , Hamdan FF , Blau N , Burlina A , Tartaglia M and Leuzzi V (2019) Neurotransmitter trafficking defect in a patient with clathrin (CLTC) variation presenting with intellectual disability and early-onset parkinsonism. Parkinsonism & Related Disorders 61, 207–210. 30337205 10.1016/j.parkreldis.2018.10.012

[ref23] Marques D , Vaziri N , Greenway SC and Bousman C (2025) DNA methylation and histone modifications associated with antipsychotic treatment: a systematic review. Molecular Psychiatry 30, 296–309. 39227433 10.1038/s41380-024-02735-x

[ref24] Mattila PK and Lappalainen P (2008) Filopodia: molecular architecture and cellular functions. Nature Reviews Molecular Cell Biology 9, 446–454. 18464790 10.1038/nrm2406

[ref25] Matus A (2000) Actin-based plasticity in dendritic spines. Science 290, 754–758. 11052932 10.1126/science.290.5492.754

[ref26] Miyamoto Y , Yamada K and Yoneda Y (2016) Importin α: a key molecule in nuclear transport and non-transport functions. Journal of Biochemistry 160, 69–75. 27289017 10.1093/jb/mvw036

[ref27] Moller HJ (2003) Amisulpride: limbic specificity and the mechanism of antipsychotic atypicality. Progress in Neuro-Psychopharmacology and Biological Psychiatry 27, 1101–1111. 14642970 10.1016/j.pnpbp.2003.09.006

[ref28] Nabais Sá MJ , Venselaar H , Wiel L , Trimouille A , Lasseaux E , Naudion S , Lacombe D , Piton A , Vincent-Delorme C , Zweier C , Reis A , Trollmann R , Ruiz A , Gabau E , Vetro A , Guerrini R , Bakhtiari S , Kruer MC , Amor DJ , Cooper MS , Bijlsma EK , Barakat TS , Van Dooren MF , Van Slegtenhorst M , Pfundt R , Gilissen C , Willemsen MA , De Vries BBA , De Brouwer APM and Koolen DA (2020) De novo CLTC variants are associated with a variable phenotype from mild to severe intellectual disability, microcephaly, hypoplasia of the corpus callosum, and epilepsy. Genetics in Medicine 22, 797–802. 31776469 10.1038/s41436-019-0703-y

[ref29] Narayana YV , Gadgil C , Mote RD , Rajan R and Subramanyam D (2019) Clathrin-mediated endocytosis regulates a balance between opposingsignals to maintain the pluripotent state of embryonic stem cells. Stem Cell Reports 12, 152–164. 30554918 10.1016/j.stemcr.2018.11.018 PMC6335602

[ref30] Nesvizhskii AI (2014) Proteogenomics: concepts, applications and computational strategies. Nature Methods 11, 1114–1125. 25357241 10.1038/nmeth.3144 PMC4392723

[ref31] Newcomer JW (2005) Second-generation (atypical) antipsychotics and metabolic effects: a comprehensive literature review. CNS Drugs 19, 1–93. 10.2165/00023210-200519001-00001 15998156

[ref32] Nganou G , Silva CG , Gladwyn-Ng I , Engel D , Coumans B , Delgado-Escueta AV , Tanaka M , Nguyen L , Grisar T , De Nijs L and Lakaye B (2018) Importin-8 modulates division of apical progenitors, dendritogenesis and tangential migration during development of mouse cortex. Frontiers in Molecular Neuroscience 11, 234. 30042658 10.3389/fnmol.2018.00234 PMC6048241

[ref33] Obri A , Ouararhni K , Papin C , Diebold ML , Padmanabhan K , Marek M , Stoll I , Roy L , Reilly PT , Mak TW , Dimitrov S , Romier C and Hamiche A (2014) ANP32E is a histone chaperone that removes H2A.Z from chromatin. Nature 505, 648–653. 24463511 10.1038/nature12922

[ref34] Panizzutti B , Bortolasci CC , Spolding B , Kidnapillai S , Connor T , Truong TT , Liu ZS , Hernández D , Gray L , Kim JH , Dean OM , Berk M and Walder K (2025) Effect of antipsychotics on the focal adhesion pathway. World Journal of Biological Psychiatry 26, 1–7. 10.1080/15622975.2025.2453181 39846496

[ref35] Pannone L , Muto V , Nardecchia F , Di Rocco M , Marchei E , Tosato F , Petrini S , Onorato G , Lanza E , Bertuccini L , Manti F , Folli V , Galosi S , Di Schiavi E , Leuzzi V , Tartaglia M and Martinelli S (2023) The recurrent pathogenic Pro890Leu substitution in CLTC causes a generalized defect in synaptic transmission in Caenorhabditis elegans. Frontiers in Molecular Neuroscience 16, 1170061. 37324589 10.3389/fnmol.2023.1170061 PMC10264582

[ref36] Park SW , Seo MK , Cho HY , Lee JG , Lee BJ , Seol W and Kim YH (2011) Differential effects of amisulpride and haloperidol on dopamine D2 receptor-mediated signaling in SH-SY5Y cells. Neuropharmacology 61, 761–769. 21663752 10.1016/j.neuropharm.2011.05.022

[ref37] Pennypacker KR (1995) AP-1 transcription factor complexes in CNS disorders and development. Journal of The Florida Medical Association 82, 551–554. 7561734

[ref38] Perrault G , Depoortere R , Morel E , Sanger DJ and Scatton B (1997) Psychopharmacological profile of amisulpride: an antipsychotic drug with presynaptic D2/D3 dopamine receptor antagonist activity and limbic selectivity. Journal of Pharmacology and Experimental Therapeutics 280, 73–82. 8996184

[ref39] Rivière JB , Van Bon BW , Hoischen A , Kholmanskikh SS , O‘roak BJ , Gilissen C , Gijsen S , Sullivan CT , Christian SL , Abdul-Rahman OA , Atkin JF , Chassaing N , Drouin-Garraud V , Fry AE , Fryns JP , Gripp KW , Kempers M , Kleefstra T , Mancini GM , Nowaczyk MJ , Van Ravenswaaij-Arts CM , Roscioli T , Marble M , Rosenfeld JA , Siu VM , De Vries BB , Shendure J , Verloes A , Veltman JA , Brunner HG , Ross ME , Pilz DT and Dobyns WB (2012) De novo mutations in the actin genes ACTB and ACTG1 cause Baraitser-Winter syndrome. Nature Genetics 44, 440–444. 22366783 10.1038/ng.1091 PMC3677859

[ref40] Schreiber J , Végh MJ , Dawitz J , Kroon T , Loos M , Labonté D , Li KW , Van Nierop P , Van Diepen MT , De Zeeuw CI , Kneussel M , Meredith RM , Smit AB and Van Kesteren RE (2015) Ubiquitin ligase TRIM3 controls hippocampal plasticity and learning by regulating synaptic γ-actin levels. Journal of Cell Biology 211, 569–586. 26527743 10.1083/jcb.201506048 PMC4639863

[ref41] Shipley MM , Mangold CA and Szpara ML (2016) Differentiation of the SH-SY5Y human neuroblastoma cell line. Journal of Visualized Experiments 2016, 53193. 10.3791/53193 PMC4828168 26967710

[ref42] Stefanelli G , Makowski CE , Brimble MA , Hall M , Reda A , Creighton SD , Leonetti AM , TaB Mclean , Zakaria JM , Baumbach J , Greer CB , Davidoff AM , Walters BJ , Murphy PJ and Zovkic IB (2021) The histone chaperone Anp32e regulates memory formation, transcription, and dendritic morphology by regulating steady-state H2A.Z binding in neurons. Cell Reports 36, 109551. 34407406 10.1016/j.celrep.2021.109551 PMC8422973

[ref43] Truong TTT , Bortolasci CC , Kidnapillai S , Spolding B , Panizzutti B , Liu ZSJ , Kim JH , Dean OM , Richardson MF , Berk M and Walder K (2022) Integrative analyses of transcriptomes to explore common molecular effects of antipsychotic drugs. International Journal of Molecular Sciences 23, 7508. 35886854 10.3390/ijms23147508 PMC9325239

[ref44] Tuncay IO , Parmalee NL , Khalil R , Kaur K , Kumar A , Jimale M , Howe JL , Goodspeed K , Evans P , Alzghoul L , Xing C , Scherer SW and Chahrour MH (2022) Analysis of recent shared ancestry in a familial cohort identifies coding and noncoding autism spectrum disorder variants. NPJ Genomic Medicine 7, 13. 35190550 10.1038/s41525-022-00284-2 PMC8861044

[ref45] Usnich T , Becker LF , Nagel I , Bäumer T and Münchau A (2024) Partially levodopa-responsive Parkinsonism in a carrier of a novel pathogenic CLTC variant. Movement Disorders Clinical Practice 11, 749–750. 38586890 10.1002/mdc3.14037 PMC11145102

[ref46] Volpon L , Culjkovic-Kraljacic B , Osborne MJ , Ramteke A , Sun Q , Niesman A , Chook YM and Borden KL (2016) Importin 8 mediates m^7^G cap-sensitive nuclear import of the eukaryotic translation initiation factor eIF4E. Proceedings of the National Academy of Sciences USA 113, 5263–5268. 10.1073/pnas.1524291113 PMC4868427 27114554

[ref47] Vontell R , Supramaniam VG , Davidson A , Thornton C , Marnerides A , Holder-Espinasse M , Lillis S , Yau S , Jansson M , Hagberg HE and Rutherford MA (2019) Post-mortem characterisation of a case with an ACTG1 variant, agenesis of the corpus callosum and neuronal heterotopia. Frontiers in Physiology 10, 623. 31231230 10.3389/fphys.2019.00623 PMC6558385

[ref48] Wagner EF (2001) AP-1 – Introductory remarks. Oncogene 20, 2334–2335. 11402330 10.1038/sj.onc.1204416

[ref49] Wang Y-Y , Hsu S-H , Tsai H-Y , Cheng F-Y and Cheng M-C (2022) Transcriptomic and proteomic analysis of CRISPR/Cas9-mediated ARC-knockout HEK293 cells. International Journal of Molecular Sciences 23, 4498. 35562887 10.3390/ijms23094498 PMC9101110

[ref50] Wei Y , Li L , Wang D , Zhang CY and Zen K (2014) Importin 8 regulates the transport of mature microRNAs into the cell nucleus. Journal of Biological Chemistry 289, 10270–10275. 24596094 10.1074/jbc.C113.541417 PMC4036152

[ref51] Weinmann L , Höck J , Ivacevic T , Ohrt T , Mütze J , Schwille P , Kremmer E , Benes V , Urlaub H and Meister G (2009) Importin 8 is a gene silencing factor that targets argonaute proteins to distinct mRNAs. Cell 136, 496–507. 19167051 10.1016/j.cell.2008.12.023

[ref52] Willner P (1997) The dopamine hypothesis of schizophrenia: current status, future prospects. International Clinical Psychopharmacology 12, 297–308. 9547131 10.1097/00004850-199711000-00002

[ref53] Wolf ME and Abi-Dargham A (2023) Synaptic plasticity as a therapeutic target to modulate circuits in psychiatric disorders. Neuropsychopharmacology 48, 1–2. 36168046 10.1038/s41386-022-01458-3 PMC9700855

[ref54] Yang C , Zheng C , Zhuang Y , Xu S , Li J and Hu C (2025a) Synaptic vesicle-related proteins and ubiquilin 2 in cortical synaptosomes mediate cognitive impairment in vascular dementia rats. Molecular Neurobiology 62, 1415–1432. 38990251 10.1007/s12035-024-04327-w

[ref55] Yang H , Sun W , Li J and Zhang X (2025b) Epigenetics factors in schizophrenia: future directions for etiologic and therapeutic study approaches. Annals of General Psychiatry 24, 21. 40186258 10.1186/s12991-025-00557-x PMC11969811

[ref56] Yao X , Chen X , Cottonham C and Xu L (2008) Preferential utilization of Imp7/8 in nuclear import of smads. Journal of Biological Chemistry 283, 22867–22874. 18519565 10.1074/jbc.M801320200 PMC2504884

