# Supplementary material for: Transcriptomic and proteomic analyses of SH-SY5Y neuroblastoma cells treated with amisulpride
Source: Acta Neuropsychiatr. 2025 Sep 16;37:e87. doi: 10.1017/neu.2025.10040 (PMC13130325; doi:10.1017/neu.2025.10040)
Supplement: Hu et al. supplementary material 1 — Hu et al. supplementary material [file S0924270825100409sup001.pdf]

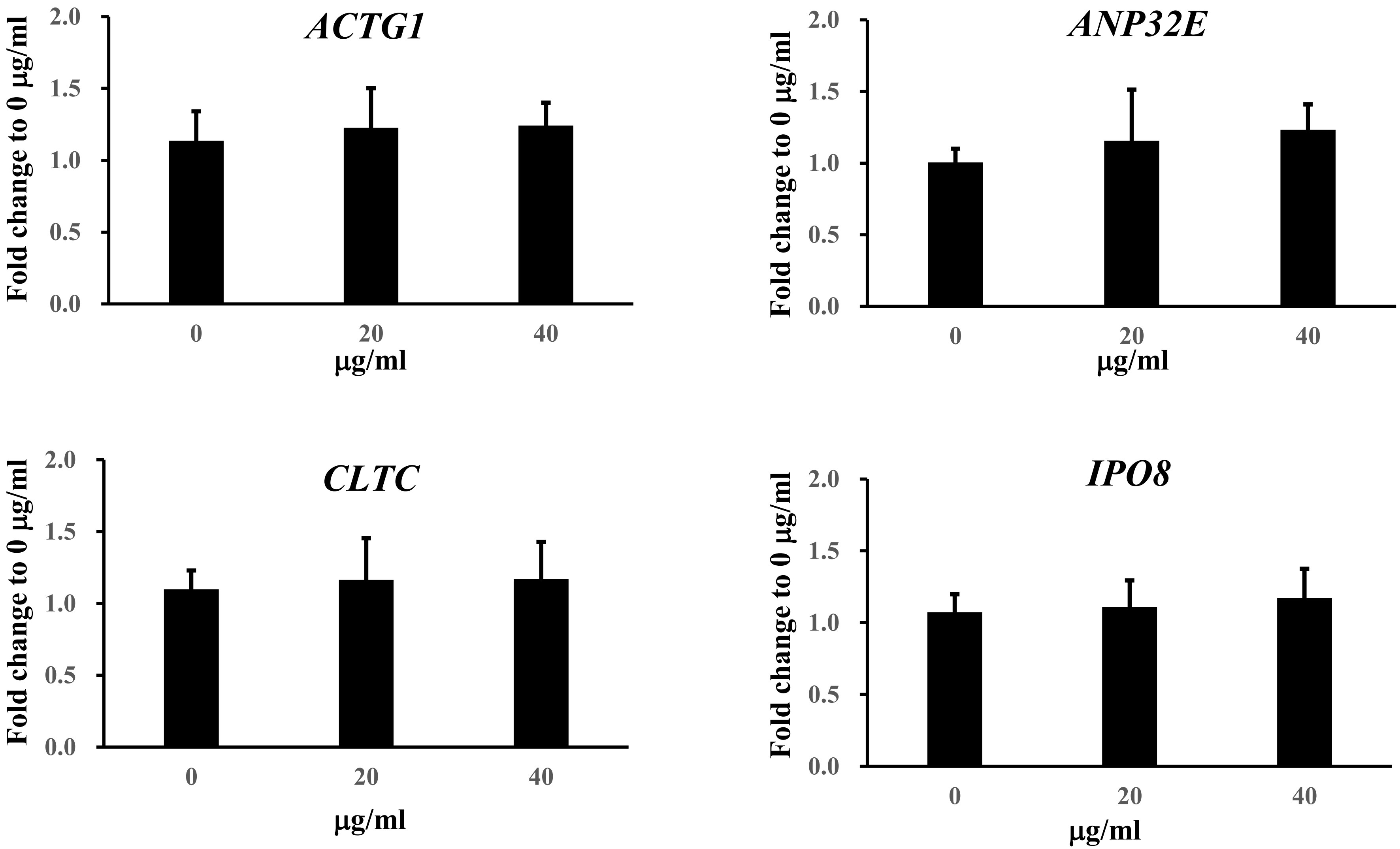
ACTG1
ANP32E
CLTC
IPO8
Fold change to 0 μg/ml
2.0
1.5
1.0
0.5
0.0
0
20
40
μg/ml

Supplementary Figure S1. RT-qPCR assay showing the expression of four genes (*ACTG1, ANP32E, CLTC*, and *IPO8*) in amisulpride-treated and non-treated SH-SY5Y cells. The *18S* gene was used as the endogenous gene for normalization. The data are expressed as fold change to the 0 μg/ml group ± standard deviation (n=3)
