# Supplementary material for: Transcriptomic and proteomic analyses of SH-SY5Y neuroblastoma cells treated with amisulpride
Source: Acta Neuropsychiatr. 2025 Sep 16;37:e87. doi: 10.1017/neu.2025.10040 (PMC13130325; doi:10.1017/neu.2025.10040)
Supplement: Hu et al. supplementary material 2 — Hu et al. supplementary material [file S0924270825100409sup002.doc]

**Supplementary Table S1.** Primer sequences, GenBank accession number, optimal annealing temperature, and length of the amplicon of genes as assayed in RT-qPCR.

| Genes | GenBank No. | Primer Sequences (5` to 3`) | | Size (bps) | Ta (^o^C) |
| --- | --- | --- | --- | --- | --- |
|  |  | Sense | Antisense |  |  |
| *ACTG1* | NM_001614 | GTGTTTCCTTCCATCGTCGG | ATGGGGTACTTCAGGGTCAG | 125 | 60 |
| *ANP32E* | NM_030920 | GATAATGAAGCGCCGGACTC | TCCTCATATCCTTCCGGTGG | 104 | 60 |
| *CLTC* | NM_004859 | TTTGTTTTGCAGTTCGGGGC | ACATCCACTGCCTTCTTTGG | 103 | 60 |
| *IPO8* | NM_006390 | GATCGCCAGCAAATACGTGA | GGCCAGTGACCAGGAAAATC | 122 | 60 |

Ta, optimal annealing temperature
