# Supplementary material for: Transcriptomic and proteomic analyses of SH-SY5Y neuroblastoma cells treated with amisulpride
Source: Acta Neuropsychiatr. 2025 Sep 16;37:e87. doi: 10.1017/neu.2025.10040 (PMC13130325; doi:10.1017/neu.2025.10040)
Supplement: Hu et al. supplementary material 3 — Hu et al. supplementary material [file S0924270825100409sup003.docx]

| **Sample** | **Total Reads** | **Unmapped** | **Total Mapped** | **Multiple Mapped** | **Uniquely Mapped** | **Splice reads （UM）** | **Non-splice reads （UM）** |
| --- | --- | --- | --- | --- | --- | --- | --- |
| Amisulpride 0 ug/ml_1 | 38707688 (100 %) | 399103 (1.03 %) | 38308585 (98.97 %) | 3316362 (8.57 %) | 34992223 (90.4 %) | 22955170 (59.3 %) | 12037053 (31.1 %) |
| Amisulpride 0 ug/ml_2 | 42673786 (100 %) | 441585 (1.03 %) | 42232201 (98.97 %) | 3234140 (7.58 %) | 38998061 (91.39 %) | 25989252 (60.9 %) | 13008809 (30.48 %) |
| Amisulpride 0 ug/ml_3 | 43162926 (100 %) | 427410 (0.99 %) | 42735516 (99.01 %) | 5660905 (13.12 %) | 37074611 (85.89 %) | 24801200 (57.46 %) | 12273411 (28.44 %) |
| Amisulpride 20 ug/ml_1 | 48933394 (100 %) | 440590 (0.9 %) | 48492804 (99.1 %) | 5860420 (11.98 %) | 42632384 (87.12 %) | 28121675 (57.47 %) | 14510709 (29.65 %) |
| Amisulpride 20 ug/ml_2 | 45314136 (100 %) | 424908 (0.94 %) | 44889228 (99.06 %) | 3808053 (8.4 %) | 41081175 (90.66 %) | 27142536 (59.9 %) | 13938639 (30.76 %) |
| Amisulpride 20 ug/ml_3 | 44943616 (100 %) | 430135 (0.96 %) | 44513481 (99.04 %) | 5702571 (12.69 %) | 38810910 (86.35 %) | 26135734 (58.15 %) | 12675176 (28.2 %) |
| Amisulpride 40 ug/ml_1 | 51321892 (100 %) | 487700 (0.95 %) | 50834192 (99.05 %) | 7490660 (14.6 %) | 43343532 (84.45 %) | 28773840 (56.07 %) | 14569692 (28.39 %) |
| Amisulpride 40 ug/ml_2 | 46223942 (100 %) | 481071 (1.04 %) | 45742871 (98.96 %) | 5127686 (11.09 %) | 40615185 (87.87 %) | 26921347 (58.24 %) | 13693838 (29.62 %) |
| Amisulpride 40 ug/ml_3 | 49243232 (100 %) | 510464 (1.04 %) | 48732768 (98.96 %) | 5562161 (11.3 %) | 43170607 (87.67 %) | 28705731 (58.29 %) | 14464876 (29.37 %) |

**Supplementary Table S2**. RNA read summary of SH-SY5Y cells treated with indicated concentration of amisulpride libraries.
